# Interactions between N‐linked glycosylation and polymerisation of neuroserpin within the endoplasmic reticulum

**DOI:** 10.1111/febs.13517

**Published:** 2015-10-03

**Authors:** Claudia Moriconi, Adriana Ordoñez, Giuseppe Lupo, Bibek Gooptu, James A. Irving, Rosina Noto, Vincenzo Martorana, Mauro Manno, Valentina Timpano, Noemi A. Guadagno, Lucy Dalton, Stefan J. Marciniak, David A. Lomas, Elena Miranda

**Affiliations:** ^1^ Department of Biology and Biotechnologies ‘Charles Darwin’Sapienza University of RomeItaly; ^2^ Department of MedicineUniversity of CambridgeCambridge Institute for Medical ResearchUK; ^3^ Department of ChemistrySapienza University of RomeItaly; ^4^ Division of Asthma, Allergy and Lung BiologyKing's College LondonUK; ^5^ Wolfson Institute for Biomedical ResearchUniversity College LondonUK; ^6^ National Research Council of ItalyInstitute of BiophysicsPalermoItaly; ^7^ Pasteur Institute – Cenci Bolognetti FoundationSapienza University of RomeItaly

**Keywords:** conformational disease, neurodegeneration, protein aggregation, serpin, serpinopathies

## Abstract

The neuronal serpin neuroserpin undergoes polymerisation as a consequence of point mutations that alter its conformational stability, leading to a neurodegenerative dementia called familial encephalopathy with neuroserpin inclusion bodies (FENIB). Neuroserpin is a glycoprotein with predicted glycosylation sites at asparagines 157, 321 and 401. We used site‐directed mutagenesis, transient transfection, western blot, metabolic labelling and ELISA to probe the relationship between glycosylation, folding, polymerisation and degradation of neuroserpin in validated cell models of health and disease. Our data show that glycosylation at N157 and N321 plays an important role in maintaining the monomeric state of neuroserpin, and we propose this is the result of steric hindrance or effects on local conformational dynamics that can contribute to polymerisation. Asparagine residue 401 is not glycosylated in wild type neuroserpin and in several polymerogenic variants that cause FENIB, but partial glycosylation was observed in the G392E mutant of neuroserpin that causes severe, early‐onset dementia. Our findings indicate that N401 glycosylation reports lability of the C‐terminal end of neuroserpin in its native state. This C‐terminal lability is not required for neuroserpin polymerisation in the endoplasmic reticulum, but the additional glycan facilitates degradation of the mutant protein during proteasomal impairment. In summary, our results indicate how normal and variant‐specific N‐linked glycosylation events relate to intracellular folding, misfolding, degradation and polymerisation of neuroserpin.

**Abbreviations**
A1AT: alpha‐1 antitrypsin
BFA: brefeldin A
DMEM: Dulbecco's modified Eagle's medium
ERAD: ER associated degradation
ER: endoplasmic reticulum
FBS: fetal bovine serum
FENIB: familial encephalopathy with neuroserpin inclusion bodies
GAPDH: glyceraldehyde‐3‐phosphate dehydrogenase
HRP: horseradish peroxidase
MD: molecular dynamics
NS: neuroserpin
RMSF: root mean square fluctuations
tPA: tissue plasminogen activator
UPR: unfolded protein response

Neuroserpin (NS) (*SERPINI1*) is a member of the serpin superfamily of serine protease inhibitors. It was originally identified as a protein secreted from neurons, but later described in other tissues outside the nervous system. The physiological roles of NS have been characterised primarily in relation to synaptic plasticity and the regulation of the neuro‐vascular compartment, both through interaction with the tissue plasminogen activator (tPA) protease (for reviews see 1, 2). NS exerts its inhibitory activity on tPA by the classical serpin mechanism, by which the reactive centre loop at the top of the serpin molecule acts as bait for the protease; upon cleavage, the protease remains covalently attached to the serpin and gets translocated to the opposite pole of the complex, suffering a distortion that inactivates its active site (reviewed in 3). To date, six point mutations have been described that lead to destabilisation and aberrant polymerisation of mutant NS within the endoplasmic reticulum (ER), causing a fatal neurodegenerative dementia called familial encephalopathy with neuroserpin inclusion bodies (FENIB): S49P and S52R 4, H338R and G392E 5, G392R 6, and L47P 7. The first four mutant variants have been shown to polymerise and accumulate within the ER in cell culture models of FENIB. The rate of polymerisation correlates with the phenotype observed in FENIB patients, with faster polymerisation being associated with earlier onset of disease 8, 9. Mutant NS is partially degraded by rapid ER associated degradation (ERAD), suggesting that it is targeted for degradation shortly after synthesis, but can also become trapped within long‐lived polymers 8, 10, 11, 12, 13.

The sequences of human and mouse NS share three predicted asparagine (N)‐linked glycosylation sites, N157, N321 and N401 14, 15, and the presence of N‐glycosylation has been confirmed by expression of human NS in multiple cell types 8, 9, 10, 11, 16. This appears to be important for the quality control of NS maturation, with mutant NS variants being directed to ERAD through interactions with the lectin OS‐9, in a manner dependent upon glycan chains at positions N157 and N321 16.

N‐linked glycosylation is also known to be important for the folding and stability of many glycoproteins 17, although little is known about the role of N‐linked glycosylation in folding or polymerisation of NS. Here we investigate the crosstalk between N‐glycosylation and polymerisation of NS by analysing the consequences of mutating each of the three predicted glycosylation sites in wild type or the pathological G392E variant of NS. In our experimental systems, wild type NS was glycosylated only at N157 and N321, while a proportion of G392E NS molecules were additionally glycosylated at the N401 position. Furthermore, we show that loss of either or both of the native glycans leads to aberrant polymerisation of wild type NS, supporting a role for N‐glycosylation in the proper folding of NS and the prevention of polymer formation.

## Results

### The polymerogenic G392E variant of neuroserpin shows an additional slower migrating band related to N‐linked glycosylation that accumulates with time

**Figure 1:**
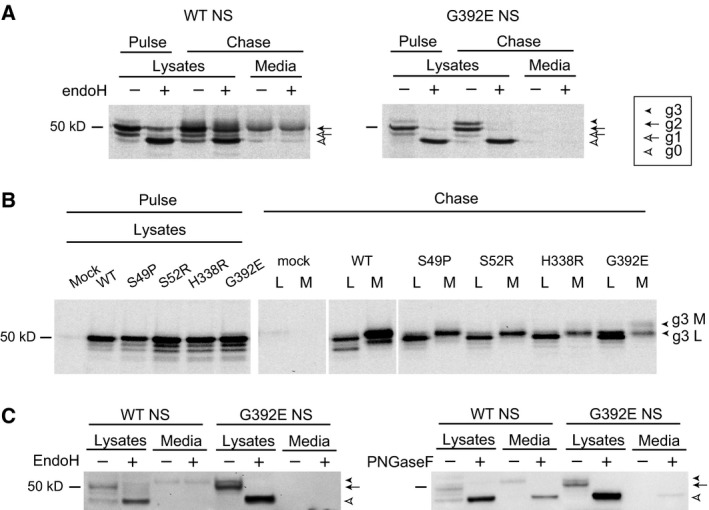
The polymerogenic G392E variant of neuroserpin migrates as a double band due to variable N‐linked glycosylation. (A) PC12 cells stably expressing wild type (WT) and G392E NS were pulsed for 20 min with [^35^S]methionine and [^35^S]cysteine and chased for 45 min. Cell lysates and culture media were treated with control buffer or endoglycosidase H (endoH), and analysed by immunoprecipitation and SDS/PAGE. White arrowheads: non‐glycosylated NS (g0); white arrow: NS with one glycan chain (g1); black arrow: main NS band with two glycan chains (g2); black arrowhead: additional slower migrating band only detectable in G392E samples (g3). Wild type NS was present in the culture medium, while G392E NS was not secreted to a detectable level. (B) COS‐7 cells transiently transfected with empty plasmid (mock), WT, S49P, S52R, H338R and G392E NS were labelled for 30 min and chased for 3 h. Immunoprecipitation analysis with an anti‐NS antibody revealed the presence of a double band only in the cell lysate (L) (g3 L black arrowhead) and culture medium (M) (g3 M black arrowhead) of cells transfected with G392E NS. (C) Cell lysates and culture media from COS‐7 cells transfected with wild type and G392E NS were digested with endoH (left panel) and peptide *N*‐glycosidase F (PNGaseF, right panel) and analysed by SDS/PAGE and western blot.

We have previously published a PC12 cell model of FENIB with inducible expression of wild type, S52R and G392E NS 9, 10. This system recapitulates key aspects of FENIB in that S52R and G392E NS show delayed secretion and accumulation of polymers within the ER. We have now assessed in further detail the nature of intracellular wild type and G392E NS in these cells by pulse‐chase labelling and endoglycosidase H (endoH) digestion. We found that wild type NS was correctly processed and secreted: enzymatic digestion of immature glycan chains with endoH led to a reduction in the molecular mass of part of the intracellular wild type NS, whereas the mature intracellular and secreted forms of the protein were not affected by the treatment (Fig. 1A, left panel). In cells expressing G392E NS, we observed a reduction of the molecular mass for all the intracellular protein upon endoH treatment (Fig. 1A, right panel), confirming its retention within the ER. No secreted G392E NS could be detected after a 45 min chase. Furthermore, we found that G392E NS showed an additional band with a slower migration (Fig. 1A, right panel, black arrowhead g3). This reported a differentially glycosylated species, consistent with a further glycosylation event, since it collapsed down to a single non‐glycosylated band upon digestion with endoH (white arrowhead g0). The extra band was minimally evident after the 20 min pulse but its intensity increased over the 45 min chase, indicating that the extra glycosylation most likely occurred after folding.

To further investigate the nature of this band we used a COS‐7 model that recapitulates the accumulation of polymers within the ER seen in FENIB 8, 9, 11, and is amenable to characterisation of new variants of NS by transient transfection. The pulse‐chase experiment in Fig. 1B shows cell lysates after a 30 min pulse and lysates and culture media after 3 h of chase of COS‐7 cells transiently transfected with human wild type, S49P, S52R, H338R and G392E NS. After the pulse, all samples showed three bands with similar patterns and intensities, supporting equal expression levels and initial glycosylation of NS in this expression system. The additional band of higher molecular mass was only detectable in the lysate and culture medium of cells expressing G392E NS (Fig. 1B, chase panel, G392E, g3 L and g3 M), suggesting that the modification leading to this band is specific for the G392E substitution. As reported before, wild type NS was detectable in the culture medium, while mutant NS showed a decrease in secretion 8, 9, 11. Using digestion with either endoH or peptide *N*‐glycosidase F (PNGaseF, which removes all types of N‐linked glycosylation) we confirmed that the additional band in lysates of cells expressing G392E NS was also due to differential glycosylation in COS‐7 cells (Fig. 1C). Wild type NS present in the culture media was resistant to endoH and sensitive to PNGaseF, as reported before for this secretory protein 8, 9.

Taken together, these results show that a variable portion of the G392E NS molecules undergoes a different pattern of N‐linked glycosylation in the ER, and that this modification is not cell‐type specific, increases with time of residence within this organelle, and is not common to other polymerogenic mutants of NS.

### Asparagines at positions 157 and 321 are glycosylated in wild type and G392E neuroserpin, while asparagine at 401 is glycosylated only in a proportion of the G392E neuroserpin molecules

**Figure 2:**
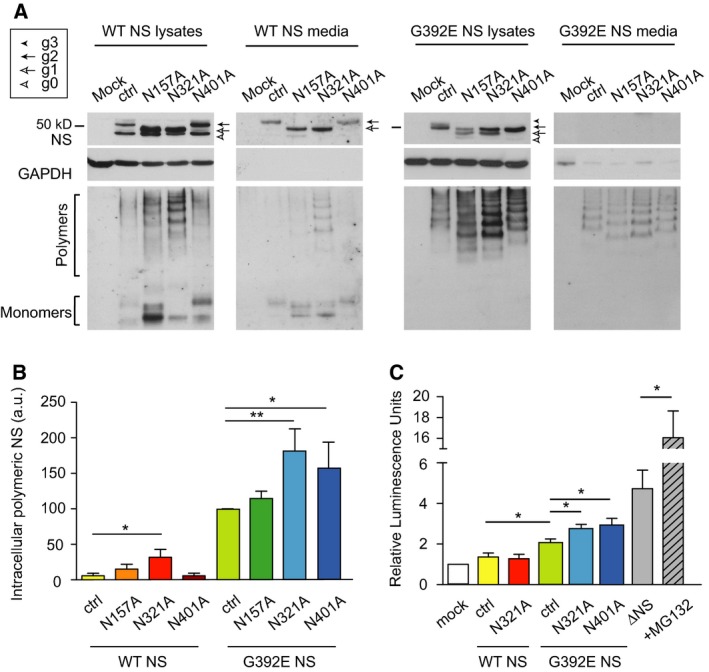
Asparagines 157 and 321 are glycosylated in wild type and G392E neuroserpin, while asparagine 401 is glycosylated only in the G392E mutant. (A) SDS and non‐denaturing PAGE and western blot analysis of lysates and culture media of cells transfected with empty vector (mock), control wild type or G392E NS (ctrl), and the different glycosylation variants on the WT or G392E NS backgrounds. Glyceraldehyde‐3‐phosphate dehydrogenase (GAPDH) served as a loading control. The differently glycosylated forms are indicated as described above for Fig. 1. Mutating N157 or N321, but not N401, to alanine caused a downward shift of wild type NS. In the case of G392E NS, the shift was observed for all three asparagines. (B) Sandwich ELISA quantification of polymeric NS in the same cell lysates as in (A), normalised to total protein and control G392E NS levels (*n* = 7). (C) ATF6 activation by luciferase reporter assay. Cells were co‐transfected with a plasmid expressing each NS variant, a plasmid encoding firefly luciferase under the control of an unfolded protein response (UPR) element, and the transfection efficiency reporter pRL‐TK *Renilla* luciferase (*n* = 6). Data are indicated as means ± SEM; Mann–Whitney test: *P < 0.05, **P < 0.01.

Since the sequence of NS has three potential glycosylation sites, we hypothesised that the additional band of slower migration reflected the glycosylation of a site normally left unglycosylated in the wild type protein. We therefore set out to characterise the usage of the three N‐linked glycosylation consensus sites: N157, N321 and N401. We mutated each glycosylation sequence from Asn to Ala, both in wild type and G392E NS. The effects were evaluated by transient transfection in COS‐7 cells and analysis of the cell lysates and culture media by SDS/PAGE and western blot (Fig. 2A, top panels). Our results showed that the substitutions at either N157 or N321 in the wild type protein caused a downward shift of the top band (Fig. 2A, wild type lysates and media panels, g2 to g1). In contrast, the N401A mutation did not cause a band shift. The N157A and N321A variants of G392E NS migrated more rapidly as doublets when compared to control G392E NS (Fig. 2A, G392E cell lysates, comparing the g3/g2 doublet with the g2/g1 doublet). The additional top band seen for G392E NS (g3) collapsed to a single band when N401 was mutated to Ala, migrating at the same size as wild type NS with two glycan chains (g2). These results indicate that wild type NS is usually glycosylated at positions N157 and N321, and that the N401 residue is glycosylated in a proportion of G392E NS molecules, appearing in SDS/PAGE as a double band composed of species with two and three glycan chains.

When the same samples were analysed by non‐denaturing PAGE, we observed that removal of the N157 or N321 glycan chains induced polymerisation of wild type NS, and polymer levels were moderately increased for all three glycosylation variants of G392E NS (Fig. 2A, lower panels). This was confirmed by quantifying the levels of intracellular polymers by sandwich ELISA, which showed a significant increase for wild type/N321A, G392E/N321A and G392E/N401A NS (Fig. 2B). It has been reported that the accumulation of polymers of Z alpha‐1 antitrypsin (A1AT) or NS within the ER does not lead to activation of the unfolded protein response (UPR) 10, 18, 19, 20, but N‐linked glycosylation is important during folding of nascent polypeptides in the ER 17. Therefore, we asked whether glycosylation mutants that lead to enhanced polymer formation activated the UPR using a highly sensitive reporter [pATF6(5X)‐Luc, also known as the pUPRE‐Luc]. COS‐7 cells were co‐transfected with this UPR reporter and the NS variants as shown in Fig. 2C. Our results showed a low activation for all the G392E NS variants when compared to wild type NS, and a small increase for the G392E/N321A and G392E/N401A variants versus control G392E NS. These signals were all low when compared to our positive control, a truncated variant of NS that undergoes misfolding in the ER (deltaNS 10), especially after proteasome inhibition by MG132. These results suggest that the lack of the glycan chains at N321 or N401 does not cause major misfolding capable of activating a robust UPR.

### Altering the normal glycosylation pattern of wild type neuroserpin leads to increased polymer formation

**Figure 3:**
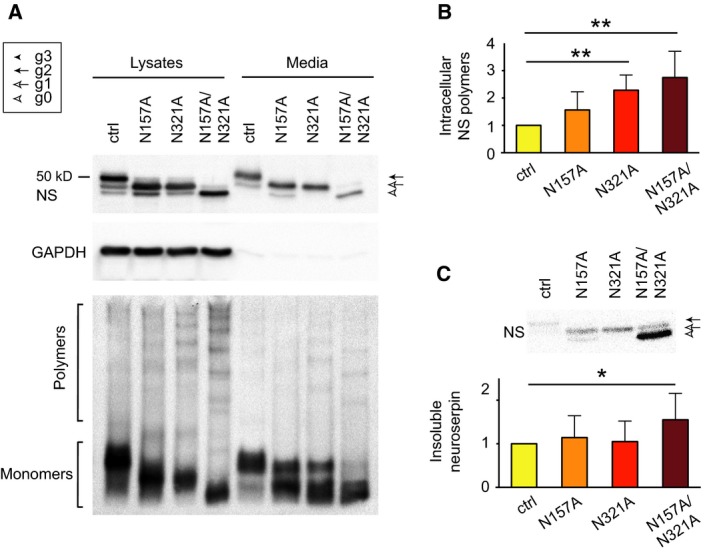
Altering the canonical glycosylation of neuroserpin leads to increased polymer formation. (A) SDS and non‐denaturing PAGE and western blot analysis of cell lysates and culture media from cells transfected with control wild type NS or the single (N157A, N321A) and double (N157A/N321A) glycosylation mutants. (B) Sandwich ELISA quantification of polymeric NS in the same cell lysates as in (A), normalised to total protein and control wild type NS levels (*n* = 5). (C) Densitometry quantification of NS in the insoluble intracellular fraction corresponding to the cell lysates shown in (A). Data are indicated as means ± SEM; Mann–Whitney test: *P < 0.05, **P < 0.01.

We sought to better understand the effects of altering the canonical glycosylation of wild‐type NS with regards to polymer formation, and created an additional wild type/N157A/N321A NS double mutant. All three glycosylation variants were assessed by SDS/PAGE and western blot of lysates and culture medium of transiently transfected COS‐7 cells. The results confirmed the presence of two glycan chains on wild type NS, one glycan on wild type/N157A and wild type/N321A NS, and no glycosylation on wild type/NS157A/N321A NS (Fig. 3A, top panel). Removal of the glycan chain at either N157 or N321 caused a small increase in polymer formation, while loss of both glycan chains led to a higher, albeit still moderate, increase that was better seen in the cell lysates (Fig. 3A, bottom panel). Quantification of polymer levels in the cell lysates by sandwich ELISA confirmed the results observed by non‐denaturing western blot (Fig. 3B). Polymer formation was correlated with the presence of NS in the insoluble fraction, as seen by densitometry quantification of NS after SDS/PAGE and western blot, particularly for wild type/N157A/N321A NS (Fig. 3C). These results highlight the role of N‐linked glycosylation in preventing aberrant polymer formation of NS within the ER.

### The pathological G392R variant of NS accumulates as polymers within the ER and does not present a slower migrating band

**Figure 4:**
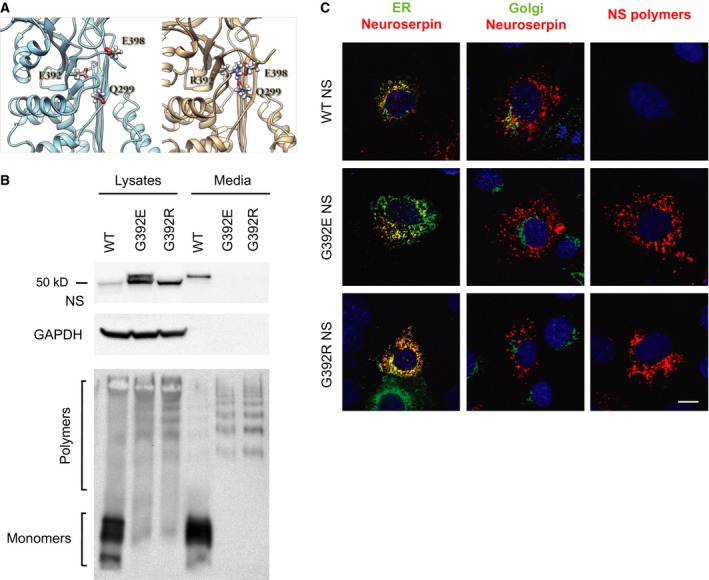
G392R NS that causes severe FENIB is retained as polymers within the ER but does not present a double band. (A) Cartoon representation of G392E NS (left) and G392R NS (right), with the interaction network of residue 392, involving E398 and Q299, shown in balls and sticks. The snapshots were chosen as the most representative of the patterns observed during the MD simulation. (B) COS‐7 cells were transiently transfected with wild type, G392E or G392R NS, and cell lysates and culture media were analysed by SDS (top) and non‐denaturing (bottom) PAGE and western blot. GAPDH served as a loading control. (C) Confocal microscopy analysis of cells transfected as in (B) and immunostained for total NS (red) and a marker for either the ER (KDEL, green) or the Golgi complex (GM‐130, green), or NS polymers alone (red). The DNA was counterstained with DRAQ5^®^. Scale bar: 10 μm.

The most recently discovered NS disease variant, G392R, is associated with the earliest age of onset of FENIB described so far 6, but its behaviour has not yet been characterised in a cellular model of disease. This variant is affected by a mutation at the same site as the G392E variant, substituting the wild type glycine (no side chain) for a residue with a long polar side chain, but of opposite charge. As position Gly392 usually folds into a hydrophobic motif within the native fold, both mutations have potential to cause local or global destabilisation. Global destabilisation can predispose to polymerisation, whereas local destabilisation will tend to do so only if remodelling of the destabilised motif is required for polymerisation 21. We undertook molecular dynamics (MD) computational modelling studies to predict the effects of these two substitutions on the structure of the native fold. The simulation indicated that in G392E NS the Glu392 would be destabilised due to repulsion by the nearby Glu398 (Fig. 4A, left panel). In contrast, in G392R NS the Arg392 forms a salt bridge with Glu398 (Fig. 4A, right panel). The latter interaction is further stabilised by the formation of a hydrogen bond between Arg392 and Gln299. Such an interaction network suggests that the G392R NS C‐terminus will be anchored to β‐sheet A, partly compensating for the destabilisation of the C‐terminus and thus reducing the accessibility of site 401 for glycosylation relative to the G392E variant.

To validate this prediction and to characterise the cellular fate of the G392R variant, we transiently transfected COS‐7 cells with wild type, G392E and G392R NS and performed SDS/PAGE and non‐denaturing PAGE analysis of cell lysates and culture media. The arginine mutant was similar to G392E NS in that it was very poorly secreted and was completely polymerised both in the cell lysate and in culture medium (Fig. 4B). Despite these similarities, G392R NS did not show a slower migrating band (g3) by SDS/PAGE. Upon immunofluorescence analysis (Fig. 4C), G392R NS was found to co‐localise with an antibody against the ER retention signal KDEL, but not with a resident protein of the Golgi compartment (GM‐130), and showed a strong polymer staining with the 7C6 monoclonal antibody (mAb) 9. These results resembled our previous findings for the well‐characterised G392E mutant of NS that causes severe dementia FENIB 9. They confirm that the pathological features of G392R NS relate to the degree of intracellular polymer accumulation of this novel variant, as seen before for the previously characterised FENIB‐causing mutations in NS 9, but not to the degree to which triple glycosylation of NS occurs.

### Retention of wild type neuroserpin within the endoplasmic reticulum does not lead to additional glycosylation of the N401 site

**Figure 5:**
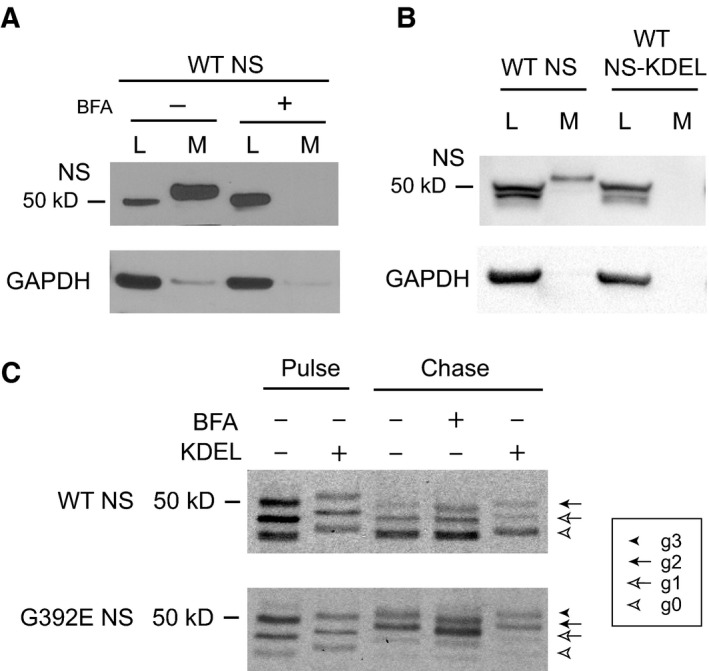
Retention of wild type neuroserpin within the endoplasmic reticulum does not lead to additional glycosylation of the N401 site. (A) COS‐7 cells were transfected with wild type (WT) NS and treated for 24 h with brefeldin A (BFA, 20 μg·mL^−1^). SDS/PAGE and western blot analysis of lysates (L) and culture media (M) showed efficient retention of WT NS within the cells. GAPDH was used as loading control. (B) COS‐7 cells were transfected with WT NS with or without a C‐terminal KDEL sequence, and cell lysates (L) and culture media (M) were analysed by SDS/PAGE and western blot. The KDEL sequence prevented secretion of WT NS into the culture medium. GAPDH was used as loading control. (C) COS‐7 cells transfected with WT or G392E NS, with or without the KDEL sequence, and treated or not with BFA as described in (A), were pulsed for 15 min with [^35^S]methionine and [^35^S]cysteine and chased for 6 h. The differently glycosylated forms are indicated as described for Fig. 1.

We have described before that G392E NS is a polymerogenic variant of NS that shows very high accumulation within the ER 9, so we were interested in understanding if the additional glycosylation chain at position N401 was added as a consequence of the prolonged residence of the mutant protein in the ER, in contrast to wild type NS that is quickly folded and secreted as mature protein. We decided to force the retention of wild type NS within the ER and evaluate its glycosylation state by SDS/PAGE and western blot analysis. COS‐7 cells transfected with wild type or G392E NS were treated with brefeldin A (BFA) that, by inhibiting normal ER–Golgi recycling, induces intracellular accumulation of secretory proteins within a compartment sharing features of both the ER and Golgi 22. To assess the efficacy of the BFA treatment, lysates of cells expressing wild type NS were collected and analysed by SDS/PAGE and western blot after 24 h of treatment. Wild type NS was completely retained within the ER upon BFA treatment, as shown by the lack of NS signal in the culture medium (Fig. 5A, top panel). G392E NS is normally absent from the culture medium in control conditions 9, 10. We next made use of metabolic labelling to optimally resolve the band patterns for detailed analysis. We analysed NS protein labelled for 15 min and chased for 6 h, as shown in Fig. 5C. Intracellular wild type NS was clearly detectable as three bands with the same molecular masses of the pulse sample, corresponding to no glycan (g0), one glycan (g1) and two glycan (g2) chains added, and the BFA treatment did not affect this pattern: no additional band was observed. Instead, G392E NS showed a fourth band after the 6 h chase, with a migration slower than the uppermost band of wild type NS, corresponding to the addition of a third glycan chain (g3), and present with similar intensities in the presence and absence of BFA. The same results were obtained when ER retention of wild type and G392E NS was forced by addition of the KDEL sequence (Fig. 5B and 5C). The third glycan chain is therefore specifically added to G392E NS, and is not just a consequence of prolonged residence of NS within the ER.

### Glycosylation of N401 facilitates the degradation of G392E NS in the context of proteasome inhibition

**Figure 6:**
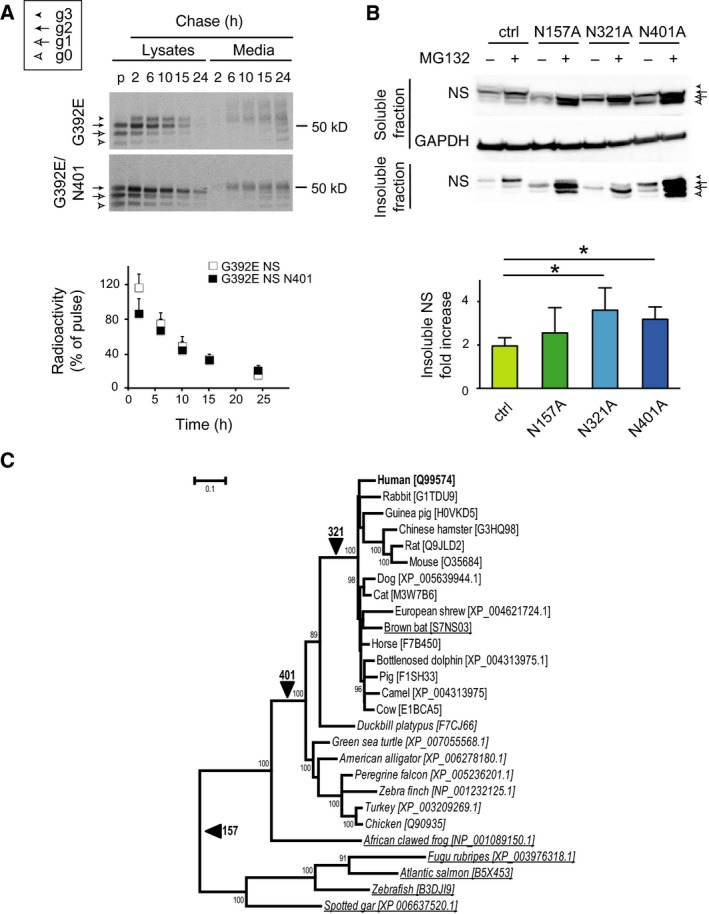
Glycosylation of N401 does not cause major changes in the intracellular handling of G392E NS, but it facilitates its proteasomal degradation. (A) COS‐7 cells transfected with G392E (top panel) or G392E/N401A (bottom panel) NS were pulsed for 15 min with [^35^S]methionine and [^35^S]cysteine and chased for the indicated times. Cell lysates and culture media were collected and NS was immunoprecipitated and analysed by SDS/PAGE. The differently glycosylated forms are indicated as described for Fig. 1. The graph shows the quantification of total intracellular NS, calculated as the addition of all the different glycosylation bands for each time point, relative to the pulse (*n* = 3). Data are indicated as means ± SEM. (B) COS‐7 cells were transfected with G392E NS in the control (ctrl), N157A, N321A and N401A versions, and treated or not with the reversible proteosomal inhibitor MG132 (2.5 μm for 24 h). NS present in the soluble cell lysates and insoluble intracellular fractions was resolved by SDS/PAGE and detected by western blot. GAPDH was used as a loading control. The graph shows the densitometry quantification of NS found in the intracellular insoluble fraction (*n* = 4), expressed as the fold increase for each variant of NS. Data are indicated as mean ± SEM; Mann‐Whitney test: *P < 0.05. (C) Codon‐aligned nucleotide sequences of neuroserpin were used to infer maximum likelihood branch lengths on a species tree derived from the NCBI taxonomy database (REF doi: 10.1093/nar/gkn741). Underlined species express neuroserpin lacking the 401 N‐linked glycosylation motif, those in italics lack the glycosylation site at 321, and all sampled sequences have the glycosylation motif at N157. Lineages along which these modifications have been acquired are indicated by arrows. Numbers at the nodes denote those with > 70% support among a set of maximum likelihood trees obtained by bootstrap resampling of the sequence alignment (*n* = 500). The tree was produced using MEGA6 39.

We next asked if the addition of an extra glycan chain at N401 of G392E NS could help to accelerate the degradation of this mutant variant, so we performed pulse–chase analysis to compare the intracellular handling of G392E/N401A with that of G392E NS in transfected COS‐7 cells. As shown in Fig. 6A, the additional band was clearly seen in cell lysates of G392E NS from the first chase time point (2 h), and from 6 h in the culture medium, but we did not detect significant differences in the behaviour of the two proteins up to 24 h of chase, suggesting that under normal conditions the proteasomal degradation of G392E NS was very efficient and was not accelerated by the presence of an extra glycan chain. To further investigate this we used the glycan‐deficient G392E point mutants and assessed their intracellular levels in the absence or presence of the reversible proteasome inhibitor MG132 (Fig. 6B). Loss of each individual glycan chain tended to increase intracellular retention relative to the wild type protein when both soluble and insoluble fractions were considered (Fig. 6B, western blot panels). This became more marked with proteasome inhibition, with significantly more insoluble G392E NS observed in the absence of glycosylation at N321 and N401 (Fig. 6B, bar graph).

### N‐linked glycosylation of neuroserpin in an evolutionary context

To provide an evolutionary context for the N‐linked glycosylation pattern of each site in NS beyond the human version of the protein, we reconstructed an evolutionary tree of NS‐expressing organisms. Nucleotide data were used to ensure discrimination of sequences at close evolutionary distances. Maximum‐likelihood branch lengths were inferred from 1230 aligned nucleotide positions, as detailed in the Experimental procedures section. This species tree provided a framework for an assessment of the evolution of N‐glycosylation patterns for NS (Fig. 6C). The glycosylation site at N157 was found to appear soon after the *SERPINI1*/*SERPINI2* gene duplication and hence is almost ubiquitous amongst the species considered. In contrast, the cryptic site at N401 was acquired later, along the lineage that gave rise to amniotes. It is thus present in the vast majority of mammals, reptiles and birds. It was later again that the glycosylation site at N321 arose, in an ancestor of placental mammals. It is notable that chicken NS has been found by SDS/PAGE and two‐dimensional PAGE to migrate in a manner consistent with occupancy of positions N157 and N401 23. From the data presented for the human orthologue, it can be speculated that later acquisition of glycosylation at N321 has resulted in a reduced occupancy at N401, and this balance is shifted in the case of G392E. Nevertheless, the high degree of retention of these positions supports a general role for glycosylation in protecting the cell against aberrant polymerisation and misfolding of NS in many species.

## Discussion

Secretory serpins such as A1AT and NS are modified by the addition of asparagine‐linked glycan chains. N‐glycosylation plays important roles in protein recognition by ER resident chaperones and other lectins for quality control during synthesis and folding, for selection of misfolded molecules for degradation by ERAD and for transport out of the ER by lectin cargo receptors 17. Point mutations can lead to serpin polymerisation and accumulation within the ER, causing a family of diseases collectively known as serpinopathies 24, 25, but the effects of N‐glycosylation on this process are largely unknown. Here, we investigated the N‐linked glycosylation of wild type and the disease mutant G392E NS, by mutating in turn the three glycosylation sites present in their protein sequence, at N157, N321 and N401, and looking at the effects on the glycosylation patterns of these proteins and their intracellular processing and polymerisation.

**Figure 7:**
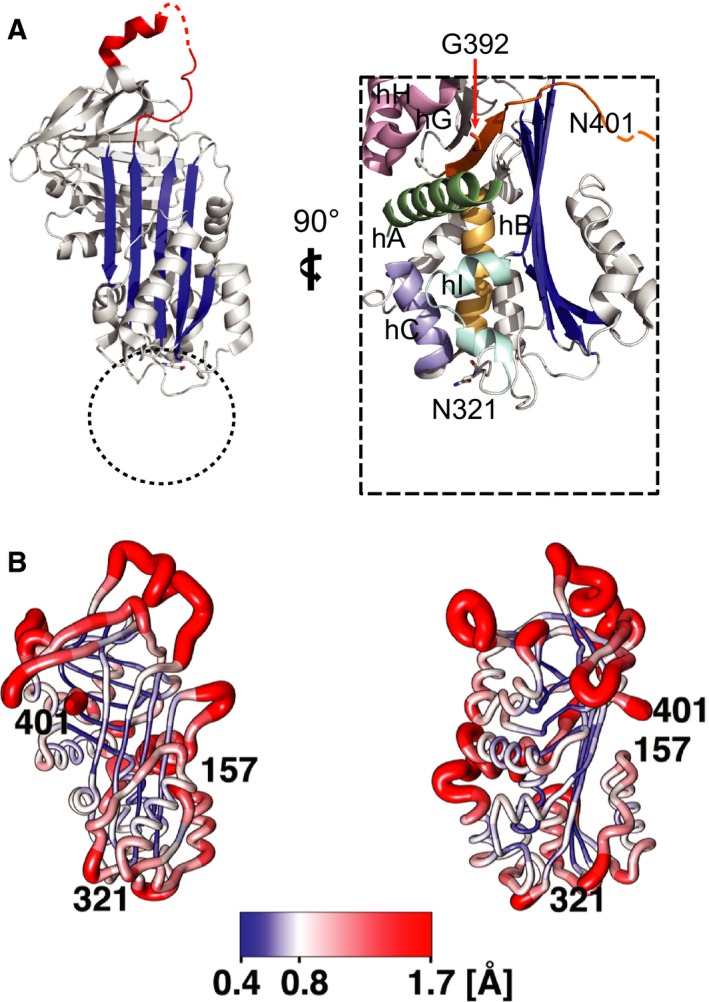
Structural detail of residue 392, and the N157, N321 and N401 (cryptic) glycosylation sites. (A) Crystal structure of native NS (PDB accession, 3FGQ) 41. The reactive site loop is shown in red, with residues undefined by electron density indicated by a dashed line; β‐sheet A is shown in blue. Zoom highlights the N321 and N401 glycosylation sites studied in this paper. The flexible glycan chain at N321 will be relatively sterically restricted by its attachment point in a loop motif sited beneath β‐sheet A and between helices B, C, and I. The dotted circle indicates the zone it likely occupies in solution, probably restricting the access of further molecules. (B) The root mean squared fluctuations averaged over each residue of wild type NS have been mapped on the structure taken from a MD simulation 35. Both the colour and the radius of the chain illustrate the changes in mobility of the molecule. The view on left is obtained by rotating the molecule by 90°. The picture was prepared using Chimera 36.

Our results show that the consensus sites at N157 and N321 of human NS are efficiently glycosylated, in the wild type protein and in all known pathogenic mutants. We observed the same effects in two different heterologous expression systems, and our results are in agreement with a recent report showing that the glycan chains at N157 and N321 of NS are important for its recognition by the lectin OS‐9, which mediates the transfer of misfolded mutant G392E NS to the ERAD machinery for degradation 16. Schipanski *et al*. 16 also showed that removal of both asparagine residues led to increased accumulation of G392E NS within the ER of HEK cells, but polymer formation was not assessed at great detail. Here we show that preventing glycosylation of wild type NS by mutating any of the sites to alanine led to mild polymerisation, higher for wild type/N321A NS, while removing both chains simultaneously caused a higher level of polymer formation. This is consistent with an evolutionary impetus to acquire and retain these sites amongst neuroserpin‐expressing vertebrates. Stability of the F‐α‐helix, which contains N157, is known to affect conformational stability of the native serpin fold 26. However, our results suggest that the glycan at N321 plays a more important role in preventing NS polymerisation. A lack of UPR activation when comparing wild type/N321A NS to the wild type protein indicates that this glycan is not required for attainment or maintenance of a globally folded state. Instead, a sugar chain at this position, oriented into solution, would exert steric effects that may limit aberrant interactions between NS molecules required for polymer assembly (Fig. 7A). The mediation of serpin conformational change by steric effects of a sugar chain has previously been reported as a regulator of the native to latent transition 27. It has also been proposed that glycan chains can favour a properly folded conformation through effects on the conformational repertoire of the unfolded state 28. An examination of the spatial distribution of root mean square fluctuations (RMSF) taken from an MD simulation of wild type NS suggests that residues in the vicinity of position 321 have an elevated mobility with respect to the bulk of the molecule (Fig. 7B). Indeed, limited proteolysis has indicated this region to be locally unfolded in the neuroserpin polymerisation intermediate 29. By contrast, the loss of the glycosylation site at position 157, which showed lower RMSF values, did not have as pronounced an effect on polymerisation. These observations are consistent with N321 glycosylation exerting a protective effect against polymer‐inducing, localised unfolding of the surrounding loop region.

The results presented here agree with previous data indicating that altering the glycosylation pattern of A1AT led to reduced secretion and to deposition of intracellular A1AT in insoluble aggregates 30. Although the conformational nature of these aggregates was not assessed, they likely corresponded to polymers of A1AT. Up to now, *in vitro* studies of the polymerisation of NS have made use of bacterially expressed recombinant protein 31. Our results point to an important role for N‐linked glycosylation in retarding polymer formation, suggesting purified glycosylated NS might provide differing results.

In our previous studies of polymerogenic mutants of NS, we observed that G392E NS expressed in COS‐7 and PC12 cells readily accumulated as polymers within the ER 9, and frequently migrated as a double band around the 55 kDa position in SDS/PAGE, both at steady state and in pulse–chase experiments. Our current results confirm that this phenotype is due to the existence of two populations of G392E molecules, with either two glycans at N157 and N321, or with three glycans at N157, N321 and N401. Our pulse–chase experiments show that G392E NS undergoes slow glycosylation of the C‐terminal asparagine, with addition of the N401 glycan probably occurring after folding. This may involve the STT3B subunit of the oligosaccharyltransferase, which can target cryptic N‐glycosylation sites 32. N401 glycosylation appears to require C‐terminal destabilisation, since it is not observed when wild type NS is retained within the ER through treatment with brefeldin‐A or addition of a KDEL motif, nor in the G392R mutant for which an electrostatic tether is suggested by molecular dynamics simulations. Interestingly, further glycosylation of N401 is also not observed in G392E NS with longer retention within the ER. This suggests that N401 glycosylation can occur soon after folding but is no longer possible over prolonged periods, during which incorporation of G392E NS into polymer chains is expected to predominate. The lack of triple glycosylation in either milder or more severely polymerogenic NS variants indicates that the C‐terminal destabilisation required for this glycosylation is not required for polymerisation. Indeed, the G392R mutation that requires the accommodation of a different large polar residue of opposite charge at the same site is strongly polymerogenic, despite predictably increasing the local stability of this folded motif. This suggests that such mutations introducing large polar side chains at this site favour polymerisation because of effects elsewhere around the molecule.

The glycosylation of a cryptic site has been described to accelerate degradation of transthyretin by transforming it from a non‐glycosylated protein into a substrate of glycoprotein ERAD 32. Our present results suggest that degradation of G392E NS is efficient when the proteasome works properly, but the presence of an extra glycan chain at N401 facilitates its degradation when the proteasome is impaired, to a similar extent to the glycan at N321, and this has been positively preserved during evolution.

In conclusion, we report here on two different roles for the N‐linked glycan chains on NS. The glycans at N157 and specially N321, present in wild type NS and all natural variants investigated here, protect against polymerisation. This effect may relate to steric impedance of intermolecular interactions or to stabilisation of a region with a tendency to increased conformational lability, particularly during polymerisation. The N401 site, present in most species, is normally unmodified, but becomes glycosylated when point mutations near to this site cause local destabilisation, and promotes degradation. N401 glycosylation therefore functions as a reporter of C‐terminal lability in NS. The fact that such glycosylation is not associated with polymerisation across any of the other disease variants studied here suggests that such C‐terminal behaviour is not critical for polymer formation in FENIB. Mutations of the glycine at position 392 to glutamic acid or arginine therefore likely cause polymerisation by destabilising interactions elsewhere in the molecule. Our results also suggest that N401 glycosylation takes place after folding, and subsequent polymerisation of G392E NS could block further modification at this site. Taken together our data advance understanding of the intracellular interplay between serpin folding, N‐linked glycosylation, degradation and polymerisation that underlies the serpinopathies *in vivo*.

## Experimental procedures

### Reagents and antibodies

Unless stated otherwise, reagents, buffers, culture media and serum for cell cultures were purchased from Sigma‐Aldrich (St Louis, MO, USA). Custom‐made rabbit polyclonal anti‐NS antibody 33 and rabbit polyclonal anti‐GAPDH antibody were from Abcam (Cambridge, UK). The mouse monoclonal anti‐NS antibodies were made in‐house as reported before 9. Anti‐KDEL was from Enzo Life Sciences (Farmingdale, NY, USA) and anti‐GM130 from BD Biosciences, San Jose, CA, USA. Goat polyclonal anti‐rabbit‐HRP (horseradish peroxidase) and rabbit anti‐mouse‐HRP are from Sigma‐Aldrich. Goat anti‐mouse IgG‐Alexa Fluor 488 and ‐Alexa Fluor 594, and goat anti‐rabbit IgG‐Alexa Fluor 594 were from ThermoFisher Scientific (Waltham, MA, USA).

### Plasmids construction

The N157A, N321A and N401A point mutations were introduced in both wild type and G392E human NS cloned in the pcDNA3.1‐myc/His plasmid (ThermoFisher Scientific) by site‐directed mutagenesis using the QuikChange XL Kit (Agilent Technologies, Santa Clara, CA, USA) following the manufacturer's protocol. In order to achieve higher levels of expression, several of the NS variants were also subcloned in the pTP6 plasmid 34, including wild type and wild type variants N157A, N321A and N157A/321A NS, and G392E and G392R NS. All plasmids were verified by sequencing. All plasmids used in each type of experiment were on the same expression vector.

### Culture of stable PC12 Tet‐On cell lines expressing neuroserpin

PC12 cells expressing wild type and G392E NS were cultured as described previously 9. Briefly, cells were maintained in Dulbecco's modified Eagle's medium (DMEM, D6546) supplemented with 10% v/v horse serum, 5% v/v Tet‐approved fetal bovine serum (FBS; BD Biosciences), l‐glutamine, 10 mm HEPES, 0.2 U·mL^−1^ bovine insulin, 200 μg·mL^−1^ Geneticin and 100 μg·mL^−1^ Hygromycin B (both selective antibiotics from ThermoFisher Scientific), at 37 °C and 10% v/v CO_2_ in a humidified incubator. NS expression was induced with 2 μg·mL^−1^ doxycycline.

### COS‐7 cells culture and DNA transfection

COS‐7 cells were maintained in DMEM (D6546) supplemented with 5% v/v FBS and Glutamax (ThermoFisher Scientific) at 37 °C and 5% v/v CO_2_ in a humidified incubator. Transfections were performed in six‐well plates or 24‐well plates with 13 mm diameter glass coverslips (for immunostaining only), treated with 0.1 mg·mL^−1^ poly‐l‐lysine. Typically, 4 μg (six‐well) or 0.8 μg (24‐well) of plasmid DNA was introduced into each well mixed with 10 μL of Lipofectamine 2000 (ThermoFisher Scientific) in serum‐free Opti‐MEM I culture medium (ThermoFisher Scientific) following the manufacturer's protocol.

### SDS and non‐denaturating PAGE and western blot analysis

The cell pellet from each well of six‐well plates was lysed in 100 μL of Nonidet lysis buffer [150 mm NaCl, 50 mm Tris‐Cl, pH 7.5, 1% v/v Nonidet P‐40, plus protease inhibitor mixture (Complete; Roche, Basel, Switzerland)]. The soluble fraction was collected in the supernatant after centrifugation at 12 000 ***g***, 4 °C for 15 min, and proteins in the insoluble pellet were extracted by heating at 95 °C in loading buffer containing 10% v/v β‐mercaptoethanol and 4% w/v SDS. Forty micrograms of total protein from each lysate and the equivalent volume of each culture medium were mixed with loading buffer as above and analysed by 10% w/v acrylamide SDS/PAGE or by BOLT 4–12% w/v precast gels (ThermoFisher Scientific) as indicated. For enzymatic digestions, 20 μg of protein and the equivalent volume of culture medium were incubated with 1000 U of endoglycosidase H (endoH) or PNglycosidaseF (PNGaseF) (both from New England BioLabs, Ipswich, MA, USA) for 3 h at 37 °C. Proteins were then separated by SDS/PAGE and analysed by western blot as described previously 8. The HRP signal was developed using the LiteAblot PLUS and TURBO extra sensitive chemoluminescent substrates (Euroclone, Pero, Italy) and exposed to film or visualised on a ChemiDoc system (Bio‐Rad Laboratories, Hercules, CA, USA).

### Metabolic labelling and immunoprecipitation

Radioactive protein labelling with [^35^S]methionine and [^35^S]cysteine and analysis by immunoprecipitation, as well as endoH analysis of radioactive samples, were performed as described before 8. Briefly, transfected cells were starved in methionine and cysteine‐free DMEM for 1 h, pulsed for 10–15 min with [^35^S]methionine and [^35^S]cysteine (1.3 MBq per well) and harvested or chased in DMEM containing 200 mm methionine and cysteine for the indicated times. After the chase, culture media were collected at 700 ***g***, 4 °C for 10 min, and the cells were harvested in Nonidet lysis buffer as above, spinning at 12 000 ***g***, 4 °C for 15 min. NS was immunoprecipitated with an anti‐NS polyclonal antibody, and immune complexes were washed and either treated with SDS/PAGE loading buffer and analysed, or treated with endoH digestion buffer (100 mm sodium citrate, pH 5.5, 1% w/v SDS, 20% v/v glycerol, 1% v/v β‐mercaptoethanol), boiled for 5 min at 95 °C and treated with 10 μL of endoH (1 munit·mL^−1^) and 1 mm phenylmethylsulfonyl fluoride (both from Sigma‐Aldrich) and incubated for 16 h at 37 °C. Radiolabelled proteins were separated on 10% w/v polyacrylamide gels, and detected by autoradiography with a Cyclone phosphor imager (Packard Instrument Co., Meriden, CT, USA).

### Sandwich ELISA

Quantification of NS in cell lysates and culture media was performed by sandwich ELISA with anti‐NS antibodies made in‐house as described previously 9. Briefly, 96‐well plates (Costar 3590; Corning Inc., New York) were coated with antigen‐purified rabbit polyclonal anti‐NS antibody (2 μg·mL^−1^), washed (0.9% w/v NaCl, 0.05% v/v Tween20) and blocked with blocking buffer (PBS, 0.25% w/v bovine serum albumin, 0.05% v/v Tween20, 0.025% w/v sodium azide). Standards (recombinant purified monomeric or polymerised NS) and samples were diluted in blocking buffer and incubated for 2 h. After washing, wells were incubated with either a pool of monoclonal antibodies (1A10 and 10B8, 0.5 μg·mL^−1^ each) or with an anti‐NS polymer monoclonal antibody (7C6, 1 μg·mL^−1^). Rabbit anti‐mouse IgG‐HRP labelled antibody was used for detection with tetramethylbenzidine substrate solution, and HRP activity was measured in a GloMax plate reader (Promega, Madison, WI, USA) at 450 nm.

### Luciferase assay

Cell transfections were performed in six‐well plates as described above. Cells were co‐transfected with 1.5 μg of each NS variant cloned in pcDNA3.1 as described above, and 1.5 μg of the reporter plasmid p(5X)ATF6‐luciferase (firefly) and 50 ng of the transfection efficiency control plasmid pRL‐TK (*Renilla*) as reported before 10. After 48 h cells were lysed in 250 μL passive lysis buffer and analysed using the Dual‐Luciferase Reporter Assay (Promega), following the recommended protocol. Both firefly and *Renilla* luciferase activities were measured using a GloMax plate reader (Promega).

### Immunofluorescence staining and confocal microscopy

COS‐7 cells were immunostained as described before 8. Briefly, 24 h after transfection cells were fixed in ice‐cold 4% paraformaldehyde, treated with blocking buffer (PBS plus 5% BSA, 0.1% Triton X‐100 and 0.1% sodium azide), and immunostained with anti‐NS rabbit polyclonal antibody or the anti‐NS polymers 7C6 mAb, anti‐KDEL and anti‐GM130 antibodies, and the corresponding secondary antibodies (goat anti‐mouse IgG‐Alexa Fluor 488 and ‐Alexa Fluor 594, and goat anti‐rabbit IgG‐Alexa Fluor 594). Nuclear DNA was counter‐stained with DRAQ5^®^ (Abcam). Coverslips were mounted with FluorSave (Calbiochem, San Diego, CA, USA) plus 2% DABCO. Imaging was performed on a Zeiss 780 confocal microscope.

### Molecular modelling

For the calculation of the RMSF of the wild type NS, we used the same MD trajectory described in 35. The reported RMSF are averages over the last 20 ns of the simulation and were mapped to a representative structure using Chimera 36. For comparative analysis of the G392E and G392R variants, two MD trajectories of 50 ns each were generated using the namd2 package and the Charm22 force field using the protocol reported in 35. The two variants were generated by using the vmd package 37, starting from a configuration obtained from a previous simulation of wild type NS 35.

### Sequence analysis

For sequence analysis, the amino acid sequence of human NS (UniProt accession Q99574) was used to search the RefSeq RNA database using tblastn (http://www.ncbi.nlm.nih.gov/blast) with an expect value cut‐off of 1.0e^−6^ but otherwise employing default parameter values. The retrieved nucleotide data was translated to amino acid data, aligned using clustalw 38, obvious misalignments manually corrected, and the nucleotide data aligned codon‐wise against this using a perl script. mega6 39 was used for phylogenetic analysis. As the desired dataset would comprise orthologous genes with high sequence identity, to increase resolution at short evolutionary distances, subsequent analyses utilised nucleotide rather than amino acid data. For each pair of sequences displaying > 97.5% identity, one representative was retained; remaining non‐NS (*SERPINI1*) sequences were removed based on an initial neighbour‐joining phylogenetic tree generated using the Tamura–Nei nucleotide substitution model. In order to explore changes in glycosylation pattern throughout neuroserpin evolution, a representative species tree was constructed based on the NCBI Taxonomy database 40. The Kimura two‐parameter substitution model, allowing rate variation across sites and accounting for invariant positions, was selected according to the Bayesian information criterion test implemented in mega6, given the tree topology and the nucleotide data. Maximum likelihood distances were accordingly inferred from the data using this model in the context of the species tree. The tree topology, based on a curated species taxonomy, was also evaluated for support with reference to a dataset of 500 maximum‐likelihood trees using boostrap resampling of the sequence alignment (same substitution model; near‐neighbour interchange heuristic).

## Author contributions

C.M. planned and performed experiments and wrote the paper; A.O. and G.L. planned and performed experiments; B.G., J.A.I. and M.M. analysed data and wrote the paper; R.N. and V.M. performed experiments, analysed data and wrote the paper; V.T., N.A.G. and L.D. performed experiments; S.M. and D.A.L. provided reagents, planned experiments and wrote the paper; E.M. planned and performed experiments, analysed data and wrote the paper.

## Conflict of interest

The authors declare no conflicts of interest.
